# A new framework for advancing in drug-induced liver injury research. The Prospective European DILI Registry

**DOI:** 10.1111/liv.15378

**Published:** 2022-07-28

**Authors:** Einar S. Björnsson, Camilla Stephens, Edmond Atallah, Mercedes Robles-Diaz, Ismael Alvarez-Alvarez, Alexander Gerbes, Sabine Weber, Guido Stirnimann, Gerd Kullak-Ublick, Helena Cortez-Pinto, Jane I. Grove, M. Isabel Lucena, Raul J. Andrade, Guruprasad P. Aithal

**Affiliations:** 1 Faculty of Medicine, University of Iceland, Reykjavík, Iceland; 2 Department of Gastroenterology, Landspitali University Hospital Reykjavik, Reykjavík, Iceland; 3 Servicios de Aparato Digestivo y Farmacología Clínica, Hospital Universitario Virgen de la Victoria, Instituto de Investigación Biomédica de Málaga y Plataforma en Nanomedicina-IBIMA Plataforma BIONAND, Universidad de Málaga, Málaga, Spain; 4 Centro de Investigación Biomédica en Red de Enfermedades Hepáticas y Digestivas (CIBERehd), Madrid, Spain; 5 Nottingham Digestive Diseases Centre, Translational Medical Sciences, School of Medicine, University of Nottingham, Nottingham, UK; 6 NIHR Nottingham Biomedical Research Centre, Nottingham University Hospitals NHS Trust and the University of Nottingham, Nottingham, UK; 7 Department of Medicine II, LMU Klinikum Munich, Munich, Germany; 8 Department of Visceral Surgery and Medicine, Inselspital University Hospital and University of Bern, Bern, Switzerland; 9 Department of Clinical Pharmacology and Toxicology, University Hospital Zurich, University of Zurich, Zurich, Switzerland; 10 Clínica Universitária de Gastrenterologia, Faculdade de Medicina de Lisboa, Universidade de Lisboa, Centro Hospitalar Lisboa Norte, HSM, Lisbon, Portugal

**Keywords:** drug aetiologies, drug-induced autoimmune-like hepatitis, drug-induced liver injury, outcomes, prospective study

## Abstract

**Background & Aims:**

No multi-national prospective study of drug-induced liver injury (DILI) has originated in Europe. The design of a prospective European DILI registry, clinical features and short-term outcomes of the cases and controls is reported.

**Methods:**

Patients with suspected DILI were prospectively enrolled in the United Kingdom, Spain, Germany, Switzerland, Portugal and Iceland, 2016−2021. DILI cases or non-DILI acute liver injury controls following causality assessment were enrolled.

**Results:**

Of 446 adjudicated patients, 246 DILI patients and 100 had acute liver injury due to other aetiologies, mostly autoimmune hepatitis (*n* = 42) and viral hepatitis (*n* = 34). DILI patients (mean age 56 years), 57% women, 60% with jaundice and 3.6% had pre-existing liver disease. DILI cases and non-DILI acute liver injury controls had similar demographics, clinical features and outcomes. A single agent was implicated in 199 (81%) DILI cases. Amoxicillin-clavulanate, flucloxacillin, atorvastatin, nivolumab/ipilimumab, infliximab and nitrofurantoin were the most commonly implicated drugs. Multiple conventional medications were implicated in 37 (15%) and 18 cases were caused by herbal and dietary supplements. The most common single causative drug classes were antibacterials (40%) and antineoplastic/immunomodulating agents (27%). Overall, 13 (5.3%) had drug-induced autoimmune-like hepatitis due to nitrofurantoin, methyldopa, infliximab, methylprednisolone and minocycline. Only six (2.4%) DILI patients died (50% had liver-related death), and another six received liver transplantation.

**Conclusions:**

In this first multi-national European prospective DILI Registry study, antibacterials were the most commonly implicated medications, whereas antineoplastic and immunomodulating agents accounted for higher proportion of DILI than previously described. This European initiative provides an important opportunity to advance the study on DILI.

## 1 Introduction

Interest in the study of *idiosyncratic* drug-induced liver injury (DILI) has increased considerably during the last two decades. Given the relative rarity of this adverse reaction, it is often not detected during drug development nor in clinical trials and becomes apparent after marketing.^1^ DILI due to troglitazone was detected late in the post-marketing phase leading to high mortality from acute liver failure among troglitazone users in the United States and other countries.^2^ The devastating consequences of troglitazone hepatotoxicity, along with severe DILI adverse reactions due to other drugs occurring pre- and post-marketing might partly explain the increased interest and funding of research initiatives in this area. In Europe, pioneering prospective studies on DILI have appeared from the Spanish DILI Registry,^3^ and other European DILI cohorts have been reported from Sweden,^4^ Iceland^5^ and Germany.^6^ Similarly, the Drug-Induced Liver Injury Network (DILIN) project, sponsored by the National Institutes of Health, has described causative agents, risk factors and outcome of DILI in the United States.^7^ In Asian countries, the more recently established Indian Network of Drug-Induced Liver Injury,^8^ as well as nationwide studies,^9,10^ reflect the growing interest in this public health burden. The European Association for the Study of the Liver (EASL)—Lancet liver commission recently highlighted DILI as an extremely challenging clinical condition due to the wide range of drugs used in clinical practice, the variety of clinical presentations and serious outcomes.^11^ The article highlighted the gaps in the DILI field, including the lack of recent information regarding its true prevalence in Europe. However, to date, no multi-national prospective cohort study of DILI has been reported from Europe.

The aim of the current study was to report the clinical presentation, drug aetiologies and outcomes from a European-wide interdisciplinary network of researchers, the Prospective European DILI (Pro-Euro-DILI) Registry.

## 2 Methods

### 2.1 Setting

The Prospective European DILI Registry (Pro-Euro-DILI), a European-wide, multicentric, prospective registry of patients with DILI and non-DILI acute liver injury controls, was established in 2016 with initial support from the European Association for the Study of the Liver (EASL). Ethical approval was obtained in each participant’s country and centre. Data collection and biobanking of biological samples were coordinated by the Biomedical Research Institute of Malaga (Spain), and the Nottingham Digestive Diseases Center of the University of Nottingham (United Kingdom), respectively. Since 2019, the Pro-Euro-DILI Registry has become a part of the DILI work package in the ‘Translational Safety Biomarker Pipeline’ (TransBioLine) Consortium project, funded by the Innovative Medicines Initiative (IMI)-2 of the European Union and the European Federation of Pharmaceutical Industries & Associations (EFPIA).^12^

### 2.2 Patients

Patients presenting with acute liver injury suspected to be due to prescription drugs, over-the-counter medications, or herbal and dietary supplements (HDS) were identified and prospectively recruited. Patients were investigated according to their individual clinical needs by clinicians in charge of patient care with an intention to secure an accurate diagnosis and appropriate management. Eligible participants were men and women aged l8 years and over, who presented with acute manifestations of liver injury and were able to give written informed consent. Potential participants who lacked capacity to give written informed consent and who had a consultee (personal or nominated) were also eligible.

Inclusion criteria were as follows: (1) to have exposure to drugs including any prescription drug, over-the-counter drug, recreational drug, herbal remedies or dietary supplements prior to the onset of liver injury; (2) to meet one of the following analytical thresholds at enrolment (day 0) and at the first sample collection visit (which in most cases was at the enrolment day): alanine transaminase (ALT) ≥5 times the upper limit of normal (ULN), alkaline phosphatase (ALP) ≥2 times ULN, or ALT ≥3 times ULN plus bilirubin exceeding 2 times ULN; (3) absence of other known causes of liver injury after detailed investigations, that can explain the acute liver injury.^13^ Patients with acute exacerbation/decompensation of known chronic liver disease that could explain the acute event were excluded. However, underlying liver disease was not a contraindication if these patients had DILI that explained their acute liver injury according to the causality assessment.

Patients aged l8 years and over, who had a drug exposure suspected to have induced the liver injury but during the diagnostic work-up were found to have acute viral hepatitis due to hepatitis A, B, C, E, cytomegalovirus (CMV), Epstein−Barr virus (EBV) or other viruses known to cause hepatitis or acute presentation of autoimmune hepatitis unrelated to the drug, ischaemic hepatitis, acute ascending cholangitis or biliary obstruction explaining cholestasis, were classified as non-DILI acute liver injury controls. Patients with unknown aetiology of the acute liver injury were excluded.

Drug-i nduced liver injury severity was graded into mild, moderate, severe or fatal/liver transplantation according to well-established criteria.^13^ Both DILI cases and non-DILI acute liver injury controls were followed up as clinically appropriate until resolution of the acute event or liver transplantation or death. The number of patients adhering to the new Hy’s law (nR ≥5 and total bilirubin >2 times ULN) was calculated, nR ratio is defined as (ALT or AST [whichever highest]/ULN) ÷ (ALP/ULN).^14^

### 2.3 Causality assessment

Formal adjudication meetings were held on a monthly basis. In these meetings, a panel of at least three clinicians (outside of the enrolling centre) with long-standing experience in clinical care of DILI patients and in DILI research reviewed all available data and ascertained whether the particular event could be adjudicated as a DILI case or as a non-DILI acute liver injury control. In particular cases, the panel of experts required further information to take a decision on a case, it was left pending and re-assessed in a later meeting.

A causal relationship between the event and the drug was assessed using the Roussel Uclaf Causality Assessment (RUCAM) scale developed by the Council of International Organization of Medical Sciences (CIOMS).^15^ Importantly, the final decision on whether the case was considered a DILI case relied on expert consensus, that is if there was discrepancy between the RUCAM scoring and the clinical judgement by the expert panel, the latter was used to determine the causality of the case.

### 2.4 Data collection

Data related to the event, clinical course, outcome and follow-up were recorded in an online Pro-Euro DILI Registry database specifically created for this project. Data retrieved include demographic characteristics, current and past medical history and drug exposure. Any medications taken in the last 6 months prior to the DILI episode were recorded, including start and stop dates. The following clinical and analytical data were entered into the database: imaging investigations such as abdominal ultrasound (US), computerized tomography (CT) and magnetic resonance cholangiography (MRC) results, liver biopsy findings, analytical data (including serological testing for viral hepatitis and presence of positive autoantibody titres and immunoglobulin G levels) corresponding to the episode.

Drug classes were classified according to the Anatomical Therapeutic Chemical (ATC) classification.

### 2.5 Statistical analysis

Variables were examined using descriptive statistics. Qualitative variables were presented using frequency distributions and compared using Pearson chi-squared test or Fisher’s exact test, as appropriate. Continuous variables were presented as mean and standard deviation (SD), or median and interquartile range (IQR). Missing values were not imputed; thus, frequencies were based on available observations. Differences between groups were assessed with the Student’s *t* test or the Mann−Whitney *U* test, or the analysis of variance (ANOVA) or the Kruskal−Wallis test, as appropriate. Tests were two-sided, a *p* value lower than.05 was deemed as statistically significant. Analyses were performed using R version 4.1.3 (R Core Team, 2013).

## 3 Results

During the study period 2016−2021, a total of 446 patients were adjudicated in the Pro-Euro-DILI Registry. Of those, 246 were adjudicated as DILI cases and 100 as non-DILI acute liver injury controls. These non-DILI cases were causally associated with acute viral hepatitis (*n* = 34), autoimmune (*n* = 41), acute biliary obstruction (*n* = 13) or other diagnoses (*n* = 12).

The remaining 100 patients (22%) were excluded in the adjudication meetings. Over half of the patients were excluded because no consensus was reached with regards to the diagnosis or because the diagnosis was inconclusive (*n* = 60). Twenty-five patients were excluded because they did not fulfil DILI criteria at the time of the first sample extraction, and 15 were due to other reasons.

### 3.1 Comparison between DILI cases and non-DILI acute liver injury controls

Differences in demographics, clinical characteristics, biochemical features and outcomes between DILI patients and non-DILI acute liver injury controls are presented in Table 1. No differences were found with regard to sex (*p* =.433) or age (*p* =.339) between these two groups. Indeed, 36% of DILI patients and 31% of non-DILI acute liver injury controls were aged ≥65 years. Non-DILI acute liver injury controls presented more frequently with jaundice (75% vs. 60% in DILI cases; *p* =.011).

Interestingly, 34% of DILI cases presented with pruritus, compared with 19% of non-DILI acute liver injury controls (*p* =.006). Somewhat unexpected, the prevalence of eosinophilia was not significantly higher in DILI cases. Non-DILI acute liver injury controls more frequently had a moderate and severe liver injury than DILI cases (*p* =.001), albeit no differences were observed in fatal outcome rates, that is liver-related death (*p* =.359) or liver transplantation (*p* =.481).

### 3.2 Comparison between aetiologies of non-DILI acute liver injury controls

The characteristics according to the aetiology of the non-DILI acute liver injury control patients are compared in Table 2. The most common diagnoses were autoimmune hepatitis (41%) and acute viral hepatitis (34%). In the latter group, viral hepatitis E accounted for almost half of the patients (*n* = 16). Patients with viral hepatitis were younger than those with other aetiologies (*p* =.015). There were differences in the pattern of liver injury between groups (*p* <.001). The vast majority of cases with autoimmune hepatitis (97%) had hepatocellular liver injury, while in patients with viral hepatitis or biliary obstruction cholestatic and mixed patterns accounted for 32% and 59% of cases, respectively. Likewise, patients with autoimmune hepatitis had more frequently jaundice (*p* =.017) and were hospitalized more often (*p* =.024) when compared with patients in the other groups. Lastly, no significant differences were found, however, a higher rate of fatal outcome in autoimmune hepatitis cases was observed (three liver-related deaths and three liver transplantations).

### 3.3 Causative drugs

A single drug was implicated in 199 cases (81%), if the fixed combination with nivolumab and ipilimumab were counted as one. The most commonly implicated conventional drugs were amoxicillin-clavulanate, flucloxacillin, atorvastatin, the combination of nivolumab/ipilimumab, infliximab and nitrofurantoin (Table 3). Conversely, there were 46 single agents, listed in Supporting Information Table S1, that were associated with only a single case each in the current study. In 37 cases (15%), two or more conventional drugs were considered to be a potential cause of liver injury, as the adjudication committee was not able to determine a single causative agent (Supporting Information Table S2).

According to the ATC classification system, the most common drug classes among single-agent cases were anti-i nfective drugs for systemic use (37%, of which nearly 90% were antibacterials) and antineoplastic and immunomodulatory agents (25%, of which one-third were immunosuppressants). In addition, drugs classified into the cardiovascular system class (mainly lipid modifying agents), and those in the nervous system class accounted for 11% and 7% of single causative agents, respectively (Table 4). In four patients, the causative agent did not have an ATC code, that is three were clinical trial drugs and the remaining was a COVID-19 vaccine (mRNA).

Patient and clinical characteristics of liver injury according to specific ATC groups and subgroups (antibacterials, cardiovascular, immunomodulators, non-steroidal anti-inflammatory drugs and central nervous system) were assessed. Hepatocellular injury predominated in all groups, ranging from 50% to 80% of cases, except in cardiovascular system drug-related cases, in which hepatocellular and cholestatic cases represented 40% each. Patients treated with immunomodulators were less likely to have jaundice or be hospitalized. Interestingly, there were very few cases that met the new Hy’s law criteria^14^ among immunosuppressants and cardiovascular groups. Indeed, no fatal cases in these groups were reported (Table 5).

A total of 18 DILI cases (7.3%) were caused by HDS, either as a single cause of liver injury or in conjunction with other agents (Supporting Information Table S3).

Comparison between main causative single agents in other different prospective DILI registries studies is shown in Table 6. Amoxicillin-clavulanate was the most common culprit in all registries, except for India, where anti-tuberculosis drugs represented nearly half of the cases. Remarkably, the vast majority of flucloxa-cillin cases (over 90%), the second common culprit in the Pro-Euro DILI Registry, were from the United Kingdom, while most of the metamizole cases came from Germany. It is also worth noting that nivolumab and ipilimumab were listed frequently as the only implicated agents in the current study, and their combination was the third most frequent culprit (along with atorvastatin), accounting for 8% of cases. The percentage of HDS was similar to the Spanish DILI Registry, but lower than in the DILIN or Indian registries, or the Icelandic study.

### 3.4 Outcome

Overall, three DILI patients (1.2%) had liver-related mortality within 6 months from presentation, while six patients (2.5%) required liver transplantation within a year, mostly within 1−2 months from DILI recognition. Hepatocellular injury predominated among these fatal/liver transplantation cases, but two of three liver-related deaths had cholestatic injury. Fatal/liver transplantation cases were caused by several different drugs. A single drug was implicated in five cases, while in three patients DILI was caused by multiple implicated drugs. The remaining liver transplant case was caused by a mixture of herbs (Table 7). In addition, three non-DILI acute liver injury controls had a liver-related death (all of them with idiopathic autoimmune hepatitis), while four patients (three of them with idiopathic autoimmune hepatitis and the remaining one with viral hepatitis E) underwent a liver transplantation.

In addition, markers of severity across prospective DILI registries were compared. The proportion of women was similar in the Pro-Euro-DILI and DILIN registries, but higher than in the Spanish and Indian registries. The hepatocellular injury was more frequent in the Pro-Euro-DILI Registry, but patients were less likely to have jaundice. In addition, the frequency of patients with pre-existing liver disease was lower when compared to the DILIN and Spanish DILI Registry (Table 8).

### 3.5 Drug-induced autoimmune-like hepatitis (DI-AILH)

Thirteen cases (5.3%) were found to have a DI-AILH phenotype. Infliximab and nitrofurantoin were responsible for five cases each. The remaining cases were due to methyldopa, minocycline and methylprednisolone. Ten cases were treated with corticosteroids and one patient continued under long-term immunosuppressant treatment, with a favourable outcome. Among those who were not treated with corticosteroids, one patient died (non-liver-related) and one underwent a liver transplantation. No relapses have been reported for any case during variable follow-up that ranged from 1 to 6years (Supporting Information Table S4).

## 4 Discussion

This is the first manuscript reporting the findings of the first multicentric prospective DILI registry in Europe. The overarching goal of the Pro-Euro-DILI Registry is to obtain biological samples from well-characterized DILI patients and non-DILI acute liver injury controls in order to discover and validate biomarkers that might assist in the diagnosis and prognostication of DILI. Therefore, a non-DILI acute liver injury control group of patients was included presenting with acute liver injury, initially suspected to have DILI but found to have another specific cause of liver injury. Interestingly, while there was no significant difference in age, sex and pattern of liver injury between DILI cases and non-DILI acute liver injury controls, a substantially higher proportion (34%) of DILI patients complained of itching compared to non-DILI acute liver injury controls (19%). Therefore, history of itching in the context of acute liver injury should raise the suspicion of drug aetiology especially when biliary obstruction is excluded by imaging. Itching may also account partly for reduced quality of life in DILI patients.^16^ It is of note that the recently revised electronic version of the RUCAM, the so-called RECAM (Revised Electronic Causality Assessment Method)^17^ does not include additional points for this feature, but future revisions of this tool may consider the evidence generated from the current study.

The aetiology of acute liver injury in the non-DILI acute liver injury control group unsurprisingly included *idiopathic* autoimmune hepatitis, acute viral hepatitis, initially unsuspected acute biliary obstruction, ischaemic hepatitis and liver injury associated with sepsis. These aetiologies are in line with the most common differential diagnoses of patients with acute liver injury in the DILIN study.^7^ Approximately 50% of patients with viral hepatitis, had hepatitis E, which is in agreement with other studies illustrating the importance of this differential diagnosis in the diagnostic work-up in all patients suspected of DILI.^18–20^

In the current study, a single drug (or over-the-counter medication) was implicated in 81% of cases, while multiple conventional causative agents were implicated in 15% of cases. These findings are in line with those reported in the DILIN registry (18%),^21^ similar to 14% in the Spanish DILI Registry^3^ but higher than the 9% in the Icelandic study.^5^

In previous prospective studies, antibacterials have dominated as the most common aetiology.^3,5,7,14,21^ In the large DILIN study, this was particularly pronounced with nine antibacterials as the most commonly implicated agents of all DILI episodes.^7^ In the current study, only three antibiotics were among the top 10 implicated agents but 40% were due to antibiotics, compared with 45% due to antibacterials in the DILIN study.^7^

In addition, 27% of cases in the Registry were caused by antineo - plastic and immunomodulating agents, which is a higher prevalence of cases when compared to other prospective registries.^7^ Thus, it seems that there has been a change in the aetiology of DILI, at least in Western countries, with a decreasing proportion of antibacterials and an increase in checkpoint inhibitors, such as the combination of ipilimumab and nivolumab. Checkpoint inhibitors have emerged as an important cause of DILI in recent years and are used more frequently.^22–25^ Furthermore, the most common immunomodulatory agents leading to DILI, were infliximab and azathioprine. Infliximab has been identified as a common cause of liver injury, belonging to category A drugs, with more than 50 DILI cases reported in the literature.^26^ Several studies have recently described the clinical characteristics and outcome in patients with liver injury due to infliximab,^27–29^ but infliximab cases have been limited in many other large cohorts of DILI patients.^3,7,14^ Interestingly, serum bilirubin levels were elevated in only 11.7% of infliximab-associated liver injury cases in this study, similarly to those previously described.^29^

All the drugs causing DILI in at least two cases have well-documented hepatotoxicity and most have been frequent causes in previous DILI studies.^3,5,7,8^ However, not all drugs were equally distributed among the participating centres in the Pro-Euro-DILI Registry. For example, flucloxacillin, a well-recognized cause of liver injury in Sweden^4^ and the United Kingdom,^30^ is not marketed in many European countries. This was reflected in fact more than 90% of the flucloxacillin cases in the current study came from the United Kingdom and the rest from Portugal. Another drug, metamizole was frequently implicated as cause of DILI, mostly together with other drugs. These DILI cases came almost exclusively from Germany, and recent studies from Germany have also showed convincingly that this old drug can be hepatotoxic.^31–33^ Another interesting difference compared with other DILI studies, was the high proportion of patients with DILI due to atorvastatin, the latter being the third most common cause of DILI. It constituted 8.6% of the cases induced by single prescription drugs, compared with only 0.9% in the DILIN study.^7^ Atorvastatin and simvastatin hepatotoxicity have been reported to be of similar frequency,^26,34^ but atorvastatin appears to cause DILI more frequently than simvastatin in more recent studies.^35,36^ Another discrepancy between causes of DILI in the United States and Europe was the low frequency of azithromycin in the current study with only two cases (0.8%) versus 2% in the DILIN study.^7^

Only 7.3% of cases in the Registry were caused by HDS (either alone or together with conventional drugs). This is lower than in the Icelandic (16%), DILIN (16%) and Indian study (14%), but similar to the figures from the Spanish registry (6%).^3,5,7,15^ In the DILIN study, HDS was the second most common group after antibacterials, whereas in the current study HDS (including anabolic steroids) were the fifth most frequent group.

Thirteen cases were found that fulfilled the proposed criteria for DI-AILH. All of the implicated agents, infliximab,^29^ nitrofurantoin,^37,38^ methyldopa,^7^ minocycline^37,38^ and methylprednisolone have been found to induce an autoimmune-like phenotype with the presence of autoantibodies and/or elevated immunoglobulin G, as well as liver histology compatible with autoimmune hepatitis. This unusual phenotype of DILI with autoimmune features seems to be more common in unselected patients with autoimmune hepatitis and was reported to occur in 9% of autoimmune hepatitis patients at the Mayo Clinic, mostly due to nitrofurantoin and minocycline.^37^ In a study from the DILIN group, clinical characteristics and autoimmune features were analysed in patients with liver injury caused by nitrofurantoin, minocycline, methyldopa and hydralazine.^38^

The prognosis of DILI patients in the current study was generally favourable, and comparable with data from other registries.^4,7,8^ The differences in prognosis between the current study and the DILIN study are unclear. It seems that our European patients had less severe liver disease, as 60% presented with jaundice vs. 70% in the US study. However, there might be several other explanations: only 9% of our patients had diabetes mellitus type II (DM) versus 25% among the US DILI patients, and pre-existing liver disease was only present in 3.2% vs. 10% in the DILIN study.^7^ Moreover, high mortality among the DILIN and Indian patients^7,8^ was associated with concomitant Stevens-Johnson Syndrome (SJS) and toxic epidermal necrolysis (TEN), whereas these severe cutaneous reactions were not observed among the DILI patients in the current study. Interestingly, there was a large difference in severity among the different drug classes. A total of 78% of patients with DILI due to antibiotics presented with jaundice, but only 13% among patients with DILI associated with immunosuppressants and no deaths in the latter group. Thus, DILI due to immunosuppressants seems to be more benign compared to other drug classes, which has also been observed in other studies.^22–24,28,29^

Management of patients with DILI was for the vast majority of cases symptomatic. In patients with acute liver failure due to DILI, emergency liver transplantation was undertaken in those without a contraindication for a liver transplant. There is currently no evidence-based pharmacological treatment that can change the natural course of liver injury in these patients as illustrated in three recent reviews on studies with ursodeoxycholic acid, n-acetylcysteine,^39^ and corticosteroids,^40^ as well as a meta-analysis of studies on prevention and management of DILI.^41^ In clinical guidelines from the European Association for the study of the liver^42^ and the American College of Gastroenterology,^43^ patients with DI-AILH should be given corticosteroids if they do not show spontaneous improvement. In the current study, 10/13 (77%) required corticosteroids, with normalization of liver tests in all and no signs of relapse after prolonged follow-up.

This study has some strengths such as being prospective, having had causality assessment by hepatologists with long-standing experience in assessing DILI patients. Importantly, it is the first European DILI registry with a standardized methodology and adjudication process with the aim to reduce the bias in diagnosis and causality assessment. However, this study also has limitations that include diagnostic insecurity due to the lack of specific biomarker for the diagnosis of DILI.

In summary, this manuscript reports the findings from the first multicentric European prospective DILI Registry. Our data confirm that antibacterials are the most common type of drugs leading to DILI, whereas antineoplastic and immunomodulating agents account for a much higher proportion of DILI than previously described. The higher proportion of DILI patients manifesting itching compared to non-DILI acute liver injury controls could be used to refine current diagnostic tools. The lesser occurrence of jaundice, diabetes mellitus and underlying liver disease in the current study as compared with other prospective DILI cohorts is an unexpected finding. This may explain why our patients had more favourable outcomes in terms of mortality and need for liver transplantation.

## Supplementary Material

Supplementary tables

## Figures and Tables

**Table 1:** Demographics, clinical features and outcome of DILI patients and non-DILI acute liver injury controls

	DILI cases (*n* = 246)	Non-DILI acute liver injury controls (*n* = 100)	*p* value
Age (years), mean±SD	56 ± 18	54 ± 18	.339
Female, *n* (%)	141 (57%)	52 (52%)	.433
Asymptomatic, *n* (%)	47 (20%)	13 (13%)	.212
Type of liver injury, *n* (%)			.094
Hepatocellular	139 (62%)	70 (75%)	
Cholestatic	42 (19%)	12 (13%)	
Mixed	44 (20%)	12 (13%)	
Jaundice, *n* (%)	141 (60%)	73 (75%)	.011
Pruritus, *n* (%)	81 (34%)	18 (19%)	.006
Eosinophilia, *n* (%)	24 (10%)	5 (5.2%)	.207
Body mass index, mean±SD	26 ± 5.0	27 ± 5.8	.297
Diabetes mellitus type II, *n* (%)	21 (9.0%)	8 (8.3%)	1.000
Dyslipidaemia, *n* (%)	41 (18%)	16 (17%)	.966
Smoking, *n* (%)	26 (11%)	21 (22%)	.019
Hospitalization, *n* (%)	169 (70%)	78 (80%)	.073
Severity, *n* (%)			.001
Mild	95 (43%)	21 (22%)	
Moderate	101 (45%)	51 (54%)	
Severe	18 (8.1%)	16 (17%)	
Fatal/liver transplantation	9 (4.0%)	7 (7.4%)	
Acute liver failure and recovery, *n* (%)	6 (2.4%)	2 (2.1%)	1.000
Liver-related death within 6 months, *n* (%)	3 (1.2%)	3 (3.1%)	.359
Liver transplantation, *n* (%)	6 (2.5%)	4 (4.2%)	.481
Non-liver-related death within 6 months, *n* (%)	3 (1.2%)	2 (2.1%)	.627

*Note:* Percentages are based on number of available observations.

**Table 2:** Demographics, clinical features and outcome in the acute non-DILI acute liver injury controls

	Acute viral hepatitis (*n* = 34)	Autoimmune hepatitis (*n* = 41)	Acute biliary obstruction (*n* = 13)	Other diagnoses^a^ (*n* = 12)	*p* value
Age (years), mean±SD	46 ± 16	58 ± 16	57± 21	58 ± 20	.015
Female, *n (%)*	12 (35%)	26 (63%)	7 (54%)	7 (58%)	.104
Asymptomatic, *n (%)*	5 (16%)	6 (15%)	2 (15%)	0 (0%)	.684
Type of liver injury, *n (%)*					<.001
Hepatocellular	22 (69%)	38 (97%)	5 (42%)	5 (45%)	
Cholestatic	4 (13%)	1 (3.0%)	2 (17%)	5 (45%)	
Mixed	6 (19%)	0 (0%)	5 (42%)	1 (9.1%)	
Jaundice, *n* (%)	20 (63%)	37 (90%)	8 (62%)	8 (73%)	.017
Pruritus, *n* (%)	6 (19%)	5 (12%)	3 (23%)	4 (36%)	.287
Eosinophilia, *n (%)*	0 (0%)	2 (4.9%)	0 (0%)	3 (27%)	.011
Body mass index, mean±SD	25±3.6	28±6.5	28±6.1	24±5.6	.107
Diabetes mellitus type II, *n* (%)	3 (9.7%)	2 (5.0%)	2 (15%)	1 (8.3%)	.562
Dyslipidaemia, *n* (%)	3 (9.7%)	10 (25%)	2 (15%)	1 (8.3%)	.349
Smoking, *n* (%)	9 (29%)	8 (20%)	1 (7.7%)	3 (25%)	.467
Hospitalization, *n* (%)	21 (66%)	38 (93%)	10 (83%)	9 (75%)	.024
Severity, *n* (%)					.236
Mild	8 (26%)	5 (13%)	4 (33%)	4 (33%)	
Moderate	18 (58%)	22 (55%)	7 (58%)	4 (33%)	
Severe	4 (13%)	7 (18%)	1 (8.3%)	4 (33%)	
Fatal/liver transplantation	1 (3.2%)	6 (15%)	0 (0%)	0 (0%)	
Liver-related death within 6 months, *n* (%)	0 (0%)	3 (7.7%)	0 (0%)	0 (0%)	.415
Liver transplantation, *n* (%)	1 (3.2%)	3 (7.7%)	0 (0%)	0 (0%)	.798
Non-liver-related death within 6 months, *n* (%)	0 (0%)	0 (0%)	1 (7.7%)	1 (8.3%)	.067

*Note:* Percentages are based on number of available observations.

a Patients with acute cholestasis, bacterial infection, cholangiocarcinoma, ischaemic hepatitis and pancreatic cancer.

**Table 3:** Most frequent single conventional drugs implicated in at least two cases

Causative agent	ATC group
Amoxicillin-clavulanate (*n* = 24)	J01CR02
Flucloxacillin (*n* = 23)	J01CF05
Atorvastatin (*n* = 16)	C10AA05
Nivolumab and ipilimumab (*n* = 16)	L01FF01/L01FX04
Infliximab (*n* = 10)	L04AB02
Nitrofurantoin (*n* = 9)	J01XE01
Disulfiram (*n* = 4)	N07BB01
Ibuprofen (*n* = 4)	M01AE01
Azathioprine (*n* = 3)	L04AX01
Diclofenac (*n* = 3)	M01AB05
Isoniazid (*n* = 3)	J04AC01
Metamizole sodium (*n* = 3)	N02BB02
Methotrexate (*n* = 3)	L01BA01
Methyldopa (*n* = 3)	C02AB01
Ribociclib (*n* = 3)	L01XE42
Rifampicin, pyrazinamide and isoniazid (*n* = 3)	J04AM05
Terbinafine (*n* = 3)	D01AE15
Azithromycin (*n* = 2)	J01FA10
Doxycycline (*n* = 2)	A01AB22
Methylprednisolone (*n* = 2)	D07AA01
Nivolumab (*n* = 2)	L01FF01

**Table 4:** Drug classes according to the Anatomical Therapeutic Chemical (ATC) classification^a^

ATC group and subgroup	*n* (%)
A (Alimentary tract and metabolism)	3 (1.5%)
B (Blood and blood forming organs)	3 (1.5%)
C (Cardiovascular system)	22 (11%)
C02 (Antihypertensives)	3 (14%)
C09 (Agents acting on the renin−angiotensin system)	1 (4.5%)
C10 (Lipid-modifying agents)	18 (82%)
D (Dermatological)	6 (3.0%)
G (Genito urinary system and sex hormones)	4 (2.0%)
J (Anti-infective for systemic use)	74 (37%)
J01 (Antibacterials for systemic use)	66 (89%)
J04 (Antimycobacterials)	7 (9.5%)
J05 (Antivirals for systemic use)	1 (1.4%)
L (Antineoplastic and immunomodulating agents)	50 (25%)
L01 (Antineoplastic agents)	33 (66%)
L04 (Immunosuppressants)	17 (34%)
M (Musculo-skeletal system)	9 (4.5%)
N (Nervous system)	14 (7.0%)
N01 (Anaesthetics)	1 (7.1%)
N02 (Analgesics)	3 (21%)
N03 (Antiepileptics)	4 (29%)
N06 (Psychoanaleptics)	2 (14%)
N07 (Other nervous system drugs)	4 (29%)
P (Antiparasitic products, insecticides and repellents)	1 (0.5%)
R (Respiratory system)	1 (0.5%)
- (Herbal and dietary supplements, including anabolic androgenic steroids)	12 (6.0%)

*Note:* Number and percentages of subgroups are calculated within ATC groups.

a Only cases with a single causative agent.

**Table 5:** Demographics, clinical data, severity and outcome of patients according to ATC classes^a^

	Antibacterials (*n* = 66)	Cardiovascular (*n* = 22)	Immunosuppressants (*n* = 17)	NSAID (*n* = 8)	CNS (*n* = 14)
Age (years), mean±SD	64 ± 12	65 ± 13	48 ± 20	42 ± 11	51 ± 9.0
Female sex, *n* (%)	42 (64%)	12 (55%)	13 (76%)	2 (25%)	7 (50%)
Type of liver injury, *n* (%)					
Hepatocellular	30 (51%)	8 (38%)	12 (75%)	5 (71%)	10 (83%)
Cholestatic	11 (19%)	8 (38%)	0 (0%)	1 (14%)	0 (0%)
Mixed	18 (31%)	5 (24%)	4 (25%)	1 (14%)	2 (17%)
Jaundice	49 (78%)	14 (64%)	2 (13%)	3 (38%)	8 (67%)
Hospitalization	39 (61%)	16 (73%)	6 (38%)	6 (75%)	9 (69%)
Rash	7 (11%)	1 (4.5%)	0 (0%)	0 (0%)	0 (0%)
Eosinophilia	13 (21%)	3 (14%)	1 (6.7%)	0 (0%)	1 (8.3%)
Lymphopenia	19 (30%)	4 (18%)	2 (13%)	2 (25%)	1 (8.3%)
Laboratory parameters at onset (×ULN), median (IQR)					
Total bilirubin	4.5 (1.9−7.0)	3.1 (1.2−5.6)	0.6 (0.5−0.8)	1.3 (0.9−3.4)	5.0 (1.0−15)
ALT	12 (7.9−19)	13 (7.0−30)	11 (9.0−18)	17 (11−32)	25 (14−35)
AST	9.5 (4.8−16)	12 (3.8−26)	10 (5.7−13)	17 (4.7−26)	16 (3.5−21)
ALP	2.3 (1.5−3.5)	3.6(2.6−6.3)	1.3 (1.0−2.7)	2.2 (1.7−2.8)	1.9 (1.3−2.2)
New Hy’s Law	18 (29%)	7 (32%)	1 (6.3%)	3 (38%)	7 (54%)
Severity					
Mild	17 (28%)	9 (41%)	11 (85%)	5 (63%)	4 (33%)
Moderate	39 (64%)	12 (55%)	0 (0%)	2 (25%)	6 (50%)
Severe	3 (4.9%)	1 (4.5%)	2 (15%)	0 (0%)	1 (8.3%)
Fatal/liver transplantation	2 (3.3%)	0 (0%)	0 (0%)	1 (13%)	1 (8.3%)

*Note*: Immunosuppressants included were infliximab, azathioprine, leflunomide, adalimumab, natalizumab, vedolizumab.

Abbreviations: ALP, alkaline phosphatase; ALT, alanine aminotransferase; AST, aspartate aminotransferase; CNS, central nervous system; IQR, interquartile range; NSAID, non-steroidal anti-inflammatory drug; SD, standard deviation; ULN, upper limit of normal.

a Only cases with a single causative agent.

**Table 6:** Most frequently implicated agents that caused drug-induced liver injury in prospective registries and studies

Pro-Euro-DILI (2016−2021) (*n* = 246)	Spanish DILI Registry (1994−2018) (*n* = 843)	DILIN (2004−2013) (*n* = 899)	Indian Network of DILI (2013−2018) (*n* = 1288)	Icelandic study (2010−2011) (*n* = 96)
Amoxicillin-clavulanate (12%)	Amoxicillin-clavulanate (22.9%)	Amoxicillin-clavulanate (10.1%)	Anti-TBC drugs (46.4%)	Amoxicillin-clavulanate (22%)
Flucloxacillin (11%)	Anti-TBC drugs (4.5%)	Isoniazid (5.3%)	Antiepileptics (8.1%)	Diclofenac (6.3%)
Atorvastatin (8.0%)	Ibuprofen (3.0%)	Nitrofurantoin (4.7%)	Non anti-TBC drugs (6.5%)	Nitrofurantoin (4.2%)
Nivolumab and ipilimumab (8.0%)	Isoniazid (2.5%)	Sulfamethoxazoletrimethoprim (3.4%)	Antimetabolites (3.8%)	Azathioprine (4.2%)
Infliximab (5.0%)	Atorvastatin (1.9%)	Minocycline (3.1%)	Anti-retroviral (3.5%)	Infliximab (4.2%)
Nitrofurantoin (4.5%)	Diclofenac (1.8%)	Cefazolin (2.2%)	NSAIDs (2.6%)	Isotretinoin (3.1%)
Disulfiram (2.0%)	Ticlopidine (1.4%)	Azithromycin (2.0%)	Hormones (2.5%)	Atorvastatin (2.1%)
Ibuprofen (2.0%)	Azathioprine (1.3%)	Ciprofloxacin (1.8%)	Statins (1.4%)	Doxycycline (2.1%)
Azathioprine, diclofenac, isoniazid, metamizole sodium, methotrexate, methyldopa, methylepitiostanol, ribociclib, anti-TBC drugs, terbinafine (1.5%)	Fluvastatin (1.3%) Simvastatin (1.3%) Paroxetine (1.2%) Nimesulide (1.1%)	Levofloxacin (1.4%) Diclofenac (1.3%) Phenytoin (1.3%) Methyldopa (1.2%)	Others (11.3%)	Imatinib (1%) Isoniazid (1%) Cefalexin (1%) Phenytoin (1%)
HDS and AAS (6.0%)	HDS and AAS (6.0%)	HDS (16.1%)	CAM (13.9%)	HDS (16%)

Abbreviations: AAS, anabolic androgenic steroids; Anti-TBC drugs, anti-tuberculosis drugs (isoniazid, rifampicin and pyrazinamide); CAM, complementary and alternative medicine; HDS, Herbal and dietary supplements; NSAID, non-steroidal anti-inflammatory drug.

**Table 7:** Characterization of DILI patients who had either liver-related death or underwent liver transplantation due to DILI

Sex and age	Causative drug(s)	Duration of treatment (days)	Type of liver injury	Bilirubin at presentation/maximal (mg/dL)	INRat onset	Outcome	Time from DILI onset to death/transplant (days)
Male, 41 years	Ibuprofen	5	Hepatocellular	12.6/18.7	1.8	Transplantation	15
Female, 59years	Azithromycin	3	Hepatocellular	9.4/23.6	2.2	Transplantation	128
Female, 44years	Trimipramine	32	Hepatocellular	16.1/17.8	2.2	Transplantation	11
Male, 7lyears	Cyclophosphamide, bortezomib	15/15	Cholestatic	19.2/26	0.9	Death	284
Male, 85years	Amoxicillin-clavulanate, azithromycin	5/2	Cholestatic	0.94/12.9	1.3	Death	18
Female, 55years	Rifampicin, pyrazinamide, isoniazid	55	Hepatocellular	10.6/29.8	2.9	Death	20
Female, 64years	Nitrofurantoin	366	Mixed	30.8/30.8	3.6	Transplantation	25
Female, 44years	Metamizole, diclofenac, ibuprofen	1/4/4	Hepatocellular	17.7/26.2	1.7	Transplantation	11
Male, 55years	Chinese herbs^a^	121	Hepatocellular	39/39	3.2	Transplantation	72

a Huang Linn, Huang Qin, Huang Bo, Zhi Zi, Sheng Di Huang, Mai Dong and Xuan Shen.

**Table 8:** Markers of severity and outcome of drug-induced liver injury in prospective registries

	Pro-Euro-DILI (2016−2021) *n* = 246	DILIN (2004−2013) *n* = 899	Spanish DILI registry (1994−2018) *n* = 843	Indian Network of DILI (2013−2018) *n* = 1288
Age (years), mean	56	49	54	43
Female sex, %	57	59	48	49
Body mass index, mean	26	27	26	22
Diabetes mellitus type II, %	9.0	25	12	6.2
Hepatocellular injury, %	62	54	57	30
Jaundice, %	60	70	69	68
Hospitalization, %	70	29^a^	60	68
Pre-existing liver disease, %	3.6	9.9	6.3	NA
Total bilirubin (mg/dL), mean	5.0	6.7	7.0	8.3
Liver-related death, *n* (%)	3 (1.2)	27 (3.0)	18 (2.1)	NA
Liver transplantation, *n* (%)	6 (2.5)	36 (4.0)	13 (1.5)	9 (0.7)^b^
Non-liver-related death, *n* (%)	3 (1.2)	29 (3.2)	14 (1.7)	158 (12.3)^c^

Abbreviations: DILI, drug-induced liver injury; DILIN, Drug-Induced Liver Injury Network; NA, data not available.

a Defined as patients with a moderate-to-severe injury who needed hospitalization.

b Personal communication Professor Harshad Devarbhavi.

c Total mortality.

## Key points

No previous multi-national study has been undertaken in Europe and results were presented on patients who have drug-i nduced liver injury (DILI). We aimed to determine the most common causes of DILI, clinical features and describe their prognosis in the United Kingdom, Spain, Germany, Switzerland, Portugal and Iceland, 2016− 2021. Amoxicillin-clavulanate, flucloxacillin, atorvastatin, nivolumab/ipilimumab, infliximab and nitrofurantoin were the most common causes of DILI.
