# Supplementary material for: A new framework for advancing in drug‐induced liver injury research. The Prospective European DILI Registry
Source: Liver Int. 2022 Aug 15;43(1):115–26. doi: 10.1111/liv.15378 (PMC7614006; doi:10.1111/liv.15378)
Supplement: Supplementary file 1 — Table S1 [file LIV-43-115-s001.pdf]

Supplementary Table 1. Conventional single drugs implicated in a single case in the Pro-Euro-DILI Registry.

Acenocoumarol
Adalimumab
Albendazole
Alendronic acid
Alverine
Amoxicillin
Ampicillin
Asparginase
Bosutinib
Carbamazepine
Celecoxib
Ciprofloxacin
Clindamycin
Clomipramine
Clopidogrel
Colesevelam
Danazol
Dextromethorphan
Estradiol
Fenofibrate
Irbesartan
Lamotrigine
Leflunomide
Levetiracetam
Minocycline
Moxifloxacin
Naproxen
Natalizumab
Ombitasvir, Paritaprevir, Ritonavir, Dasabuvir
Pazopanib
Pembrolizumab
Piperacillin
Pivmecillinam
Pregabalin
Propofol
Pyrazinamide
Rivaroxaban
Sulfamethoxazole-trimethoprim
Temozolomide
Tibolone
Trabectedin
Trametinib
Trimipramine
Vedolizumab
Vincristine
Zanubrutinib

Supplementary Table 2. List of patients in whom multiple drugs were implicated in causing drug-induced liver injury.

Sex and age	Causative agents			
Female, 62y	Asparaginase	Vincristine	Daunorubicin	
Female, 48y	Diclofenac	Ibuprofen		
Female, 63y	Amoxicillin-clavulanate	Doxycycline	Amoxicillin	
Male, 38y	Mirtazapine	Amitriptyline		
Male, 66y	Aripriazole	Milnacipran		
Male, 47y	Atorvastatin	Ezetimibe		
Male, 71y	Cyclophosphamide	Bortezomib		
Female, 49y	Methylprednisolone	Ocrelizumab		
Male, 85y	Amoxicillin-clavulanate	Azithromycin		
Male, 35y	Isotretinoin	Atorvastatin		
Female, 31y	Ibuprofen	Metamizole		
Female, 60y	Amoxicillin-clavulanate	Azithromycin		
Female, 21y	Methylprednisolone	Ethinylestradiol		
Male, 30y	Levofloxacin	Ibuprofen		
Female, 58y	Rifampicin	Moxifloxacin		
Male, 55y	Ciprofloxacin	Metronidazole	Paracetamol	
Female, 74y	Dapsone	Docetaxel		
Female, 74y	Trazodone	Fluoxetine		
Female, 44y	Metamizole	Diclofenac	Ibuprofen	
Female, 58y	Pregabalin	Metamizole	Naproxen	
Male, 23y	Metronidazole	Ceftriaxone	Paracetamol	
Male, 36y	Moxifloxacin	Ceftriaxone		
Male, 69y	Amoxicillin-clavulanate	Clarithromycin		
Female, 55y	Osimertinib	Valproic acid		
Female, 51y	Acemetacin	Rivaroxaban		
Female, 79y	Amoxicillin-clavulanate	Levofloxacin	Rifampicin	
Male, 82y	Pioglitazone	Metamizole		
Female, 74y	Flucloxacillin	Metronidazole		
Male, 44y	Metamizole	Ibuprofen	Diclofenac	Tizanidine
Female, 64y	Metamizole	Clindamycin		
Female, 39y	Isoniazid	Rifampicin	Levofloxacin	
Female, 38y	Amoxicillin-clavulanate	Metamizole		
Female, 57y	Ribociclib	Letrozole		
Female, 78y	Capecitabine	Gemcitabine		
Male, 19y	Metamizole	Pantoprazole		
Female, 74y	Azathioprine	Doxycycline		
Male, 57y	Pembrolizumab	Axitinib		

y: years

Supplementary table 3. Herbal and dietary supplements and anabolic androgenic steroids that were implicated in causing drug-induced liver injury*.

**Causative agents (n=18)**	**Type**
Methylepitiostanol (n=3)	AAS
Methylstenbolone	AAS
Androstan derivate	AAS
Whey protein supplement (for bodybuilding purpose)	HDS
Clenbuterol and fat burner product	HDS
Weight loss supplements (n=2)	HDS
Iberogast† (n=2)	HDS
Chinese Herbs∫	HDS
Supplement with *Chelidonium*, *Echinacea* and *Lycopodium*	HDS
Supplement with Kaloba and *Pelargonium*	HDS
Green tea extract	HDS
Chinese tea	HDS
Supplement with curcuma	HDS
Mixed herbal-based supplement	HDS

AAS: anabolic androgenic steroids; HDS: herbal and dietary supplements

* Either single or multiple drugs.

† Combination of herbs, such as celandine, angelica.

∫ Huang Lim, Huang Qin, Huang Bo, Zhi Zi, Sheng Di Huang, Mai Dong, and Xuan Shen.

Supplementary table 4. Demographic and clinical characteristics of drug-induced autoimmune-like hepatitis cases.

Sex and age	Drug	Indication	Latency	ANA	SMA	High IgG	Corticosteroids	IS at follow-up	Relapse	Biopsy	Follow-up
Female, 44y	Infliximab	Crohn's disease	4 months (4 infusions)	1:6400	Negative	NA	Yes	No	No	None	6y
Female, 68y	Infliximab	Ulcerative colitis	3 months (4 infusions)	1: 200	NA	NA	No	No	No	None	6y
Male, 28y	Infliximab	Psoriasis	1.5 months (3 infusions)	1:300	Negative	No	Yes	No	No	None	2y
Female, 65y	Infliximab	Psoriasis	3 months (3 infusions)	1:300	1:160	Yes	No	No	No	None	12 months
Female, 37y	Infliximab	Psoriasis	2 months (3 infusions)	Negative	Negative	Yes	Yes	No	No	None	6y
Female, 35y	Methyldopa	Hypertension	5 months	1:3200	Negative	Yes	Yes	Yes	No	Inflammation, no fibrosis	2y
Female, 22y	Minocycline	Dermatitis	3 months	Negative	Negative	NA	Yes	No	No	None	6y
Female, 84y	Nitrofurantoin	Urinary tract infection	18 months	Positive	Negative	Yes	No	No	No	Inflammation, no fibrosis	2y
Female, 47y	Nitrofurantoin	Urinary tract infection	24 months	Negative	Negative	No	Yes	No	No	Inflammation, no fibrosis	4 months*
Male, 73y	Nitrofurantoin	Urinary tract infection	2 months	1:6400	Positive	Yes	Yes	No	No	Inflammation, no fibrosis	20 months
Female, 64y	Nitrofurantoin	Urinary tract infection	12 months	Positive	Negative	No	Yes	No	No	Inflammation, mild fibrosis	19 months†
Female, 62y	Nitrofurantoin	Urinary tract infection	15 months	1:100	Negative	Yes	Yes	No	No	None	12 months
Male, 29y	Methylprednisolone	Multiple sclerosis	6 days	1:100	Negative	No	Yes	No	No	Inflammation, no fibrosis	3y

ANA: antinuclear autoantibody; SMA: smooth muscle autoantibody; IgG: immunoglobulin G; IS: immunosuppression; y: years; NA: data not available

* Non-liver related death (COVID-19 infection).

† Liver transplantation
